# Effective strategies against COVID-19 and the importance of infection sequelae

**DOI:** 10.1186/s41256-022-00283-x

**Published:** 2022-12-01

**Authors:** Jade Khalife

**Affiliations:** Social Medicine and Global Health, Faculty of Medicine, Lund University, Jan Waldenströms Gata 35, 214 28 Malmö, Sweden

**Keywords:** COVID-19, Strategy, Sequelae, Long COVID, Chronic disease

## Abstract

COVID-19 is a serious threat to human health and development. The acute burden of the pandemic includes more than 18.2 million deaths worldwide, and is unprecedented in modern times. This represents only a fraction of the total burden, as it excludes infection sequelae. An effective global strategic paradigm has been missing throughout the pandemic. The ‘flattening the curve’ approach neglected the importance of infection sequelae, and being centered on healthcare capacity was conceptually contrary to a people-centered health system. In March 2022, the World Health Organization revised its pandemic approach, importantly shifting emphasis away from managing transmission and towards prevention. Despite limitations, this now recognizes the role of infection sequelae, whose impact is becoming clearer in both variety and scale. Drawing on the foundational concepts of Sun Tzu and Carl von Clausewitz, most country approaches do not qualify as strategies, but rather as operational plans. They are also largely ineffective, neglecting infection sequelae, viral evolution dangers and other parameters. The purpose of this article is to summarize the evidence on COVID-19 infection sequelae, and alongside other contextual parameters use this to motivate that infection should be prevented. This is then used to answer the question: What is an effective strategy against COVID-19?

## Background

The COVID-19 pandemic that has raged since 2020 has come at a high cost for both health and economy. This includes an estimated 18.2 million deaths worldwide up to December 2021, and hundreds of millions harmed [1]. Global health leadership was initially slow to respond adequately, and arguably still lags. This may be largely attributable to the challenges in the structure of global health governance, and a scarcity of expertise in responding to novel pathogenic threats.

In March 2020, the World Health Organization (WHO) called for slowing the transmission of SARS-CoV-2, by ‘flattening the curve’, to delay the epidemic peak and allow health systems to cope with demand [2]. This approach was undoubtedly more humane and protective of health than allowing widespread transmission. However, it ignored the precautionary principle towards a novel threat, particularly in terms of infection sequelae. It was also conceptually centered on health systems themselves. As such, it used hospital capacity to determine interventions, rather than the number of people infected, harmed or dying. This represented the dominance of a self-centered health system, over a people-centered one.

At the national level, authorities pursued different approaches in responding to COVID-19. Throughout 2020–21 these approaches varied across a spectrum of allowing mass-infection, through to mitigation, containment and intermittent elimination. Mass-infection, or the pursuit of herd-immunity through infection, stood out as an unethical and unscientific approach. Yet, this was pursued explicitly or implicitly in countries such as Sweden, the Netherlands and the United Kingdom [3, 4]. This most contrasted with the approach of countries such as China, New Zealand, Thailand and Vietnam, who adopted containment and intermittent elimination approaches [3]. The latter approach aims to prevent all or most transmission, usually including quarantine and isolation measures. Most countries pursued a mitigation approach to ‘flatten the curve’, which accepts increasing or high transmission, but within a threshold.

The roll out of COVID-19 vaccines in 2021 provided a several-fold reduction in the risk for hospitalization and death. However, these vaccines were not designed to provide sterilizing immunity, and viral transmission remained largely unhindered. The advent of new viral variants further challenged countries, particularly as of late 2021 with Omicron variants which possess immune-evasive properties. Re-infection with previous variants had been limited, but became common with Omicron.

Throughout 2022, numerous countries relaxed protective measures against COVID-19. Most countries that had succeeded in containing or intermittently eliminating the virus reverted to weaker mitigation approaches (e.g. New Zealand, Thailand), with China notably remaining an exception. In many instances the public was misled that Omicron was a ‘mild’ variant, which led to further transmission and pandemic harm. In some countries, such as Denmark, health authorities abandoned all mitigation measures while cases were still increasing, thus pursuing mass-infection, under presumption that infection with Omicron would provide high or ‘herd immunity’ to future variants [5]. Lacking scientific foundation, it was unsurprising that such approaches have failed to prevent subsequent surges with new Omicron sub-variants (BA.4/5).

The end of the pandemic heralded by some has failed to materialize. Policy debate and emphasis has largely been at the level of measures (e.g. surveillance, testing). Most countries’ approaches lack clarity regarding both the purpose and direction of measures. An overall framework, or strategy, is absent. Approaches have also largely neglected the mounting evidence of harmful sequelae among COVID-19 survivors, with important implications across societal health and development.

In March 2022, the WHO defined two ‘strategic objectives’: to reduce and control COVID-19 incidence; and to prevent, diagnose and treat COVID-19 [6]. It also justified the purpose for each of these, albeit with emphasis on those most vulnerable or at risk, rather than all individuals. It also notably lacks any mention of SARS-CoV-2 transmission being airborne [7]. In December 2021, WHO had recognized airborne transmission, however, it does not engage the public with this knowledge. Despite its limitations, the March 2022 update represents an important development for a global strategic paradigm to counter COVID-19. As such, the emphasis of WHO has shifted away from managing transmission and towards prevention.

In this article, I summarize the evidence on longer-term harms of COVID-19 infection, and the overall context of the pandemic and its possible trajectories. I then clarify the distinction between measures versus strategy, and conclude with a template for what an effective strategy against COVID-19 would be.

## Sequelae of COVID-19 infection

COVID-19 can be more accurately considered as a complex multi-system disease, rather than a respiratory disease. A wide body of evidence has already documented damage to various organs and systems, including the heart, brain, lungs, and kidneys. Many of these changes were already apparent in limited animal and human investigations during the first half of 2020, but have since been confirmed in numerous studies.

Long-term cardiovascular outcomes among survivors beyond 30 days after infection have included increased risk for stroke, transient ischemic attacks, atrial fibrillation, sinus bradycardia, ventricular arrhythmia, atrial flutter, acute coronary disease, myocardial infarction, ischemic cardiomyopathy, angina, heart failure, pericarditis, myocarditis, pulmonary embolism, deep and superficial vein thrombosis, and other cardiac disorders [8, 9]. Hospitalized people had greater incidence of these findings compared to non-hospitalized people. Both adult and children survivors had an increased risk of incident diabetes in the post-acute phase of COVID-19 infection [9–11]. Children who had been infected were more than twice as likely to have a subsequent diabetes diagnosis, than those not infected [12].

Nervous system impact from COVID-19 includes damage to various regions of the brain, and a wide manifestation of conditions including anosmia, encephalitis, seizures, musculoskeletal disorders, peripheral nervous system disorders and reduced cognition and memory. At 12 months the risk for neurologic disorders was increased by 42% following COVID-19 infection, which translates into 7 cases per 100 infected individuals [13]. Increased neurologic disorders were found across the age spectrum, and risk was more elevated for younger people than older people for cognitive and sensory disorders, including Guillain-Barré syndrome and encephalitis or encephalopathy [13]. The UK Biobank study used MRI of 401 people, before and after infection (mainly mild, not hospitalized), revealing the loss of grey matter, tissue damage, and reduction in brain size [14]. Investigations revealing brain hypo-metabolism among both adult and children survivors have further confirmed damage to the brain [15, 16]. Furthermore, the formation of amyloid plaques (as seen in Alzheimer’s dementia) has also been documented, with two peptides from the SARS-CoV-2 proteome found to self-assemble into amyloids [17, 18]. Neurodegenerative biomarkers have also been found to be elevated among hospitalized people to levels similar to those seen in people with Alzheimer’s dementia [19].

Mental health is also affected due to infection. Psychiatric sequelae were found to be greater among COVID-19 survivors during the 6 months following diagnosis, compared to survivors of influenza and other respiratory tract infections [20]. This included dementia, mood and anxiety disorders and psychotic disorder, and the findings were consistent among non-hospitalized and hospitalized people [20]. Survivors of severe COVID-19 infection were more likely to have long-term mental morbidity, specifically depression and anxiety [21]. Given the high similarity between SARS and SARS-CoV-2, the neurological, cardiopulmonary and mental health sequelae would be expected to be predominant among survivors [22, 23].

COVID-19 also results in auto-immunity, with various auto-antibodies being formed even following mild infection. These functional auto-antibodies are directed against body organs including lung, gastro-intestinal tract, skin and central nervous system; as well as vascular cells, coagulation factors, platelets, connective tissue; and disturb immune function and impair virological control [24]. Importantly, auto-immunity has also been shown to persist in those experiencing Long COVID, specifically with auto-antibodies against different G-protein-coupled receptors, which are known to interrupt vascular and neuronal processes [25].

Accelerated biological aging has been found among COVID-19 survivors, including those that had mild infections. This epigenetic impact increases with younger age, and is also accompanied by telomere shortening [26, 27]. Evidence also suggests that the immune system itself is damaged following COVID-19 infection, with resultant decrease in naïve T-cells, which play a critical role in response to novel pathogens [28]. As such, the former finding draws some similarity to HIV, which also results in naïve T-cell reduction.

The risk of dying from COVID-19 is recognized to increase with older age, but it also increases risk of dying among all age groups. Mortality from COVID-19 is not only in the acute phase, but also extends to the longer term. A nationwide 12-month cohort found that infection resulted in substantially increased mortality in the months after the acute infection phase had ended [29]. People that had survived a severe infection were almost three times more likely to die within a year of infection, than those not infected [30].

Several mechanisms seem to be involved in the damage caused by SARS-CoV-2 in the human body. The virus’ spike protein has high affinity for human ACE2 receptors, which is abundant in various tissues, and in particular in endothelial cells throughout the human vasculature [31]. Its neurotropic potential is also recognized, having the ability to damage nerve tissue, likely mediated by ACE2 receptors or neuropilin-1 on olfactory mucosal cells and olfactory epithelium, respectively [32, 33].

It is important to note that while vaccination is beneficial, it is not sufficiently protective against COVID-19 impact. Among survivors of the acute phase of infection, excess death at 6 months among vaccinated people was at 1.3%, compared to 2.0% among those unvaccinated [34]. This is due to several factors, most notably waning of temporary immunity conferred by vaccines (centered on neutralizing antibodies), and increased immune-evasion of recent variants. The protective effect of vaccination has been continuously eroded with new variants, particularly with the immune-evasion properties of Omicron. Even among the Omicron sub-variants, both Pfizer and Moderna recently announced that their boosters against BA.4 and BA.5 resulted in three-times lower neutralization antibodies than they had against BA.1 [35, 36].

Waning immunity and immune-evasive variants have also resulted in considerably increased reinfections, which have been more common in 2022 than throughout 2020–2021. The harms of reinfection are cumulative. A US nationwide cohort study on reinfections has found that compared with one infection, those with two or more infections had increased risk of death, hospitalization, post-COVID medical events and organ system disorders, with results being consistent among those vaccinated and non-vaccinated [37].

The patient-created term ‘Long COVID’ has been used to denote the long-term sequelae due to infection, typically after 4–8 weeks after infection. This includes a wide range of symptoms and conditions, including myalgia, neuralgia, dysautonomia, excessive fatigue, fever, skin manifestations, and shortness of breath. This can include prolonged, debilitating and chronic symptoms. By mid-2021, more than 50 long-term effects of COVID-19 had been identified [38]. While post-acute sequelae are not uncommon with other pathogens, those due to COVID-19 are numerous, and the widespread population exposure would result in a considerably large burden [39]. The incidence of Long COVID has been found to vary by study design and context, often ranging between 20 and 50% of all infections, and increasing with severity and increased age. Among children and adolescents 25% develop Long COVID following infection, including infants, and up to 58% among those hospitalized [40, 41]. The most common symptoms in this age category are mood changes, fatigue, sleep disorders, respiratory symptoms, sputum/nasal congestion and changes in cognition (concentration, learning difficulties, confusion, memory loss) [40]. Using US CDC data, a preprint study has estimated Long COVID due to the recent Omicron sub-variant BA.5 to be 22% [42].

The involvement of different mechanisms is suspected in Long COVID, including auto-immunity, superantigen-mediated activation of the immune system, occult viral persistence, endothelial dysfunction and coagulation activation [43, 44]. The repeated findings of viral persistence in diverse organs, including bone marrow, is a concerning finding whose implications will require further investigation [45, 46]. Viral persistence result in a chronic inflammatory process, and is a feature of several oncogenic viruses such as human T-cell leukemia retrovirus and Epstein-Barr virus (also implicated as the leading cause of multiple sclerosis). Preliminary investigations have also suggested plausible pathways whereby severe COVID-19 infection may cause acute and persistent reduction of p53 tumor suppressor gene, with important implications for future population health [47].

The tally and burden from Long COVID remains largely uncaptured in national and international data. Awareness on Long COVID remains very limited among health professionals and the general public. This renders many sufferers ‘invisible’ to the health system, particularly in countries not engaging the public on this issue, and lacking diagnostic capacity among its healthcare workers.

The UK Office for National Statistics provides the most consistent national record of Long COVID, with up to 20% of infected people reporting symptoms for 5 weeks or longer, and 10% symptomatic for 12 weeks or longer [48]. Among those with mild or initially asymptomatic infection 21% reported symptoms for 30 days or longer after infection. The estimated number of people with Long COVID in the UK has continued to increase throughout the period between May 2021 and May 2022, with the greatest prevalence (as proportion to total population) being among 35–69 years old [48]. Healthcare workers are also impacted, with over 10,000 NHS personnel reportedly off work for more than 3 months due to Long COVID [49]. In May 2022 the Bank of England Monetary Policy Report noted that the main factor behind the large workforce decrease (by 440,000 people) has been long-term sickness mainly due to Long COVID and the rise in NHS waiting lists [50].

## Vaccines, viral evolution and uncertainties

We can identify several parameters and uncertainties regarding the evolution of the threat posed by COVID-19. These mainly involve changes in the virus itself (new variants), development of new tools, and the human response to this threat. We can expect that improved tools for prevention and treatment will continue to be developed. These include spike-protein-based vaccines and pan-coronavirus vaccines. Still more promising would be intranasal and inhaled vaccines, which would provide protection through mucosal immunity at the site of infection, and likely high protection against transmission. Mucosal protection would bring herd immunity within reach for the first time in this pandemic. The first of such vaccines have recently been approved in China (Convidecia Air™, inhaled) and India (iNCOVACC™, intranasal); both are viral vector (non-replicating) vaccines [51, 52]. An additional 14 candidate vaccines are in clinical development across various countries [53].

There continued to be abundant speculation on the future evolution of the virus itself and our immune response, often influenced by miss or dis-information. Much of this largely fails to consider uncertainties and/or established scientific evidence. There is no selective pressure on the virus to become less virulent. The ‘law of declining virulence’ (or ‘avirulence hypothesis’) originated in the 1880s, however, this has been widely displaced by the ‘trade-off model’ and its extensions that have developed since the 1970s [54]. This latter model explains that an optimal level of virulence is determined by a range of factors, including length of time between infection and symptom onset, and host susceptibility. Throughout 2020–2021 we have seen more virulent COVID-19 variants displacing less virulent ones. Although the Omicron variant was estimated to be almost half as virulent as the Delta variant, it was more immune-evasive, which contributed greatly to its contagiousness. Unfortunately, the relaxed approach many countries took against Omicron further contributed to its spread. This resulted in Omicron (BA.1, BA.2) causing more deaths and more Long COVID worldwide than the previous Delta variant had. Furthermore, allowing regular or widespread transmission means greater viral replication, and thus greater likelihood of more problematic variants arising [55].

In July 2021 the UK Scientific Advisory Group for Emergencies (SAGE) released a report on the long-term evolution of SARS-CoV-2 [56]. The report considered various scenarios, including one of a more harmful variant comparable to SARS-CoV or MERS-CoV (10–35% case fatality), which it considered to be a realistic possibility. Three other scenarios included were a variant that evades current vaccines (‘realistic possibility’), a drug-resistant variant (‘likely’) and a variant with decreased virulence (‘unlikely in short-term’). The emergence of Omicron (immune evasion, decreased virulence versus Delta—but not the original strain) underscores the unpredictable evolution of the virus. As such, from a risk mitigation perspective, it is important to consider the implications for similar or worse future threats from COVID-19.

Our response to the COVID-19 threat is a major determinant to the harm we endure. A coordinated global strategy is necessary. However, considering the barriers limiting such a global approach, effective national strategies become all the more indispensable.

## An effective strategy

Strategy differs from operational plans or tactics. The first is intended to provide an overall framework or system, towards achieving long-term goals, typically on the scale of several months to years. The second provides specific and smaller steps, which—when tied together within an overall framework (i.e. strategy)—achieve long-term goals.

The greatest fundamental contributions to the concept of strategy come from the writings of Sun Tzu (sixth century BC) and Carl von Clausewitz (eighteenth century AD). Sun Tzu notes that “All men can see these tactics whereby I conquer, but what none can see is the strategy out of which victory is evolved” [57]. Despite strategy being a necessity, many countries failed to develop genuine strategies to counter COVID-19, and instead relied on (tactical) operational plans with various measures (e.g. contact tracing, testing).

Von Clausewitz makes a similar separation: *Zweck-Ziel-Mittel* as the Purpose-Goal-Means [58]. As such, one has to first define what is the purpose (end state) intended; the goals/intermediate goals to reach there, and the means necessary to achieve these. Therefore the purpose provides the overall framework; the goal(s) helps guide towards achieving the purpose; and the means clarifies what is needed for this. Within the health discipline it has been common to use mission-goals-objectives as equivalents to Purpose-Goal-Means. However, the latter terminology may be more widely understood.

What would an effective strategy on COVID-19 look like? First, to qualify as a strategy it would have to clearly define the Purpose-Goal-Means. Second, to be effective, these definitions should be based on current knowledge and take into account the parameters previously discussed, such as infection sequelae, protectiveness of vaccines and uncertainties of viral evolution.

**Fig. 1:**
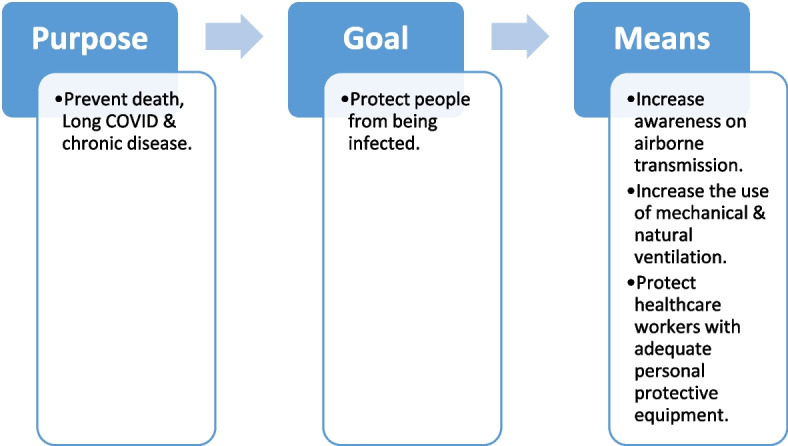
The basis of an effective strategy against COVID-19: Defining first the purpose, followed by the goal and means

Given that infection is harmful, the basis of an effective strategy would have prevention of infection sequelae and of death as a purpose; and protection from infection as a goal (see Fig. 1). The means would include increasing awareness of airborne transmission, improved ventilation, and improved protection of healthcare workers. This may be considerably expanded, for example to include increasing the capacity of healthcare professionals (means); and diagnosis and treatment of people with Long COVID (goal). An effective strategy would provide clarity, and also avoid situations where people’s well-being becomes secondary to other interests.

When approaches to counter disease fail, it is likely due to a combination of factors, including political will and technical competence. Specific gaps may be the weak capacities within the health sector for strategy development, as well as for decision-making frameworks under conditions of uncertainty. Increased investment in capacity building on these topics is important, alongside multi-sectoral and multi-disciplinary collaboration, to address both the ongoing COVID-19 pandemic and future threats.

## Conclusions

The end of the COVID-19 pandemic has not yet arrived. SARS-CoV-2 continues to claim many lives and harm many more. We should not gamble our well-being and that of future generations by leaving the initiative to viral evolution. Numerous countries failed to develop effective pandemic strategies, with approaches being unclear. Many of these did not qualify as genuine strategies. Governments have the responsibility to develop and implement effective strategies to protect their populations. This includes clear definitions of the Purpose, Goal and Means, informed by current evidence and respect for uncertainty. These strategies should put people first.

## Data Availability

No database or primary data was used in preparing the manuscript.
